# Possible associations between ELF electromagnetic fields, DNA damage response processes and childhood leukaemia

**DOI:** 10.1038/sj.bjc.6601010

**Published:** 2003-06-10

**Authors:** P Hone, A Edwards, J Halls, R Cox, D Lloyd

**Affiliations:** 1 National Radiological Protection Board, Chilton, Didcot, OX11 0RQ, UK; 2 Brunel Institute for Bioengineering, Brunel University, Uxbridge, UB8 3PH, UK

**Keywords:** electromagnetic fields, DNA repair, chromosome aberration, childhood leukaemia

## Abstract

Epidemiology has shown an association between exposure to extremely low frequency (ELF) electromagnetic fields (EMF) and childhood leukaemia. The causal nature and biological basis of this association are however questionable. Studies with aneuploid cell lines raised the hypothesis that ELF EMF may act as a coleukaemogen by compromising DNA damage response to genotoxic agents such as ionising radiation. We examined this hypothesis using *γ*-ray-induced dicentric chromosome exchange in human lymphocytes. The results from 12 h post-*γ*-ray exposure to fields of 0.23, 0.47 and 0.7 mT provide no support to the hypothesis. The power of the study was sufficient to exclude an ELF enhancement of chromosomal exchange of 10–15% (2SE).

The potential health effects of environmental exposure to extremely low frequency (ELF) electromagnetic fields of 50–60 Hz is an important, but not fully resolved, issue in public health.

Although the results of epidemiological studies on the potentially tumorigenic effects of ELF fields have been largely negative (IARC, 2002), pooled analyses provide evidence that prolonged, time-weighted average exposure above 0.4 *μ*T is associated with a small risk of leukaemia in children (Ahlbom *et al*, 2000, 2001; NRPB, 2001). These data have insufficient strength to justify firm conclusions on a causal relation and a chance finding is possible. Thus, in the absence of epidemiological replication and plausible biological mechanisms, the available ELF-childhood leukaemia data remain difficult to interpret.

Considering biological mechanisms, there is convincing evidence that ELF fields alone do not damage chromosomal DNA (Murphy *et al*, 1993), and direct leukaemogenic activity therefore, seems, unlikely. However, *in vitro* studies have sought and found preliminary evidence, in aneuploid mammalian cell lines, that ELF fields may potentiate the cellular effects of ionising radiation in the context of gene mutation induction (Walleczek *et al*, 1999) and the modification of DNA repair-related cell cycle checkpoints (Harris *et al*, 2002). Accordingly, a plausible mechanistic hypothesis would be that ELF fields possess coleukaemogenic activity centred on their flux density-dependent ability to perturb DNA damage response processes in target haemopoietic cells.

The present study sought to critically examine this hypothesis by utilising the quantitatively reliable assay of dicentric chromosomal exchanges in freshly isolated human blood lymphocytes (IAEA, 2001). The use of exchange aberrations in diploid human lymphocytes as a biological end point contributes additional relevance in respect of haemopoietic cell lineage and the known involvement of primary chromosomal exchange events in childhood leukaemogenesis (Greaves, 2002). Such balanced chromosomal translocations are induced by ionising radiation at an equal frequency with unbalanced dicentrics exchanges (Finnon *et al*, 1999), and both are formed by the same processes of misrepair of the initial DNA lesions. Mitotic nondisjunction effectively eliminates most unbalanced exchanges but, in the *in vitro* test system used here, synchronised cells were analysed in their first postirradiation metaphase. On this basis, the dicentric may be used as a surrogate for quantifying the frequency of the less easily observed translocation type exchange. The study design employed placed emphasis on controlled/blind-coded ELF exposures and the statistical resolution of potentially weak effects of ELF.

## MATERIALS AND METHODS

The ELF field exposure system used was designed specifically for cell biology studies and reduces physical and biological variables to a minimum (Wolff *et al*, 1999). It comprises two identical solenoid cells in a highly controlled environment for cell cultures (temperature, humidity and atmosphere). For each set of experiments, the choice of which coil was providing an exposure and which was the sham was computer-coded; decoding occurred when the study was complete.

In each of six experimental runs, a fresh blood sample taken, with informed consent and in accordance with the institutional ethics protocol, from a healthy 46 years female donor was cooled to ice temperature. Most of the blood sample volume was *γ* irradiated to 2 Gy (^60^Co source, dose rate 1 Gy min^−1^, dose reproducibility within 2%), while the remaining volume acted to provide unirradiated controls. Post-*γ* ray/control blood cultures in 10 ml plastic tubes comprised 0.3 ml blood plus 4.5 ml of appropriately supplemented, ice-cooled Eagles' essential medium, which included 5-bromodeoxyuridine to define the cell cycle status of metaphase cells to be scored (IAEA, 2001). All cell cultures remained at ice temperature for 2 h to allow for transport to the ELF facility. At this time, 0.1 ml mitogenic phytohaemagglutinin was added to each culture and these were then placed in the ELF rig where they warmed to 37°C. Cultures were maintained at this temperature during the 12 h exposures to ELF at flux densities of 0.23, 0.47 and 0.7 mT at 50 Hz. The six runs, each with six *γ*-irradiated and two unirradiated cultures per coil, provided duplicates of all ELF and sham exposures; ELF flux density variations were within 1%. Following 12 h ELF/sham exposure, cultures remained in the rig at 37°C for a further 36 h, thus providing a standard post-*γ*-incubation period of 48 h for mitotic development with colcemid added at 45 h (IAEA, 2001). Cultures of *γ*-irradiated cells (transport controls) were set up and processed in an identical fashion in each run, but were not taken to the ELF facility. Coded slides were prepared and 200 first division metaphase spreads per culture were scored for dicentric exchanges using internationally recommended protocols (IAEA, 2001).

## RESULTS AND DISCUSSION

Following cytogenetic scoring, the *χ*^2^ test, based upon Poisson assumptions, was used to test homogeneity of dicentric aberration yields from replicate samples. The coil and slide codes were then broken and standard errors on differences between ELF-exposed and sham-exposed samples in the same run were computed from the sum of the individual Poisson variances.

In brief, homogeneity in dicentric aberration yields was confirmed for individual cultures in each run (data not shown); Table 1

**Table 1:** Dicentric exchanges scored in lymphocytes exposed to combinations of 2 Gy *γ*-rays and three ELF flux densities with shams and controls

					**Difference between exposed and sham**	**Probability^a^ (*P*)**	
**Run**	**2 Gy** ***γ***	**EM field**	**No of cells scored**	**Dicentrics**		**One-sided**	**Two-sided**
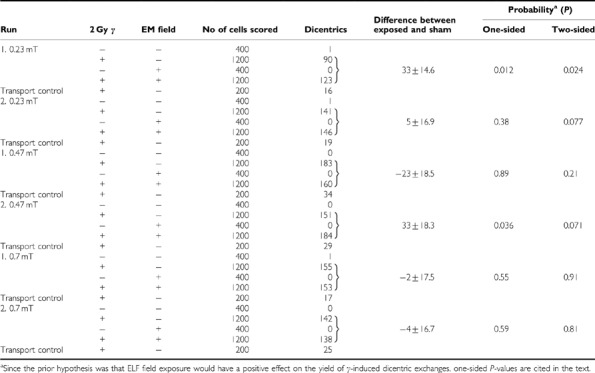							

a Since the prior hypothesis was that ELF field exposure would have a positive effect on the yield of *γ*-induced dicentric exchanges, one-sided *P*-values are cited in the text.

shows the results from the pooled replicates of the six experimental runs. In only one run, the first at 0.23 mT, was there a difference at a borderline level of significance (*P*=0.012) between dicentric aberration yields in ELF-exposed and sham-exposed cultures, that is, ELF>sham. Such a difference was not seen in the second run at 0.23 mT. Neither was there evidence of a trend towards an ELF effect on the yield of post-*γ* ray dicentric exchanges in the duplicate higher exposure runs at 0.47 and 0.7 mT. Thus, given the multiplicity of intercomparisons, the small difference seen in the first run at 0.23 mT may well be a chance finding. It is notable that the ELF sham (*γ* only) dicentric yield in this run was unexpectedly lower than in others (*P*<0.0001), and residual biological variability may provide an explanation. Allowing for differences in cells scored, the transport control data on dicentric yield are also statistically compatible with those of the ELF sham exposures and correspondingly, with no effect from ELF. In addition, the data add weight to the prevailing view that ELF fields alone do not directly damage chromosomal DNA.

In conclusion, this statistically robust experimental study provides no support for the mechanistic hypothesis that environmental ELF fields at relatively high levels might be causally associated with excess childhood leukaemia via the perturbation of chromosomal damage response processes in target haemopoietic cells. This conclusion is contingent on conditions of *γ*/ELF exposure used and the power of the study that we estimate to be sufficient to exclude an overall ELF effect of 10–15% (2SE).
